# Increased Prorenin Expression in the Kidneys May Be Involved in the Abnormal Renal Function Caused by Prolonged Environmental Exposure to Microcystin-LR

**DOI:** 10.3390/toxics12080547

**Published:** 2024-07-27

**Authors:** Yuuka Hitsuda, Yoshihito Koto, Hideaki Kawahara, Koichi Kurata, Keisuke Yoshikiyo, Kohji Nishimura, Ayumi Hashiguchi, Hideaki Maseda, Kunihiro Okano, Norio Sugiura, Kazuya Shimizu, Hidehisa Shimizu

**Affiliations:** 1 Graduate School of Natural Science and Technology, Shimane University, 1060 Nishikawatsu-Cho, Matsue 690-8504, Japan; 2 Graduate School of Life and Environmental Science, Shimane University, 1060 Nishikawatsu-Cho, Matsue 690-8504, Japan; 3 Institute of Agricultural and Life Sciences, Academic Assembly, Shimane University, 1060 Nishikawatsu-Cho, Matsue 690-8504, Japan; 4 The United Graduate School of Agricultural Sciences, Tottori University, 4-101 Koyama-Minami, Tottori 680-8553, Japan; 5 Interdisciplinary Center for Science Research, Shimane University, 1060 Nishikawatsu-Cho, Matsue 690-8504, Japan; 6 Faculty of Environmental, Life, Natural Science and Technology, Okayama University, 3-1-1, Tsushima-Naka, Kita-ku, Okayama-shi 700-8530, Japan; 7 Biomedical Research Institute, National Institute of Advanced Industrial Science and Technology, 1-8-31 Midorigaoka, Osaka 563-8577, Japan; 8 Faculty of Bioresource Sciences, Akita Prefectural University, Akita 010-0195, Japan; 9 Faculty of Life and Environmental Sciences, University of Tsukuba, Ibaraki 305-8572, Japan; 10 Faculty of Life Sciences, Toyo University, Gunma 374-0193, Japan; 11 Estuary Research Center, Shimane University, 1060 Nishikawatsu-Cho, Matsue 690-8504, Japan

**Keywords:** cyanotoxin, microcystin, prorenin, TGFβ1, α-SMA, renal fibrosis

## Abstract

Toxic algae in eutrophic lakes produce cyanotoxic microcystins. Prior research on the effect of microcystin-LR in the kidney utilized intraperitoneal injections, which did not reflect natural exposure. Oral microcystin-LR research has focused on renal function and histopathology without examining the molecular mechanisms. The present study aimed to evaluate the mechanism of microcystin-LR in the kidneys via oral administration in WKAH/HkmSlc rats over 7 weeks, alongside stimulation of the proximal tubular cells. Although there were no differences in the concentrations of plasma albumin, blood urea nitrogen, and creatinine, which are parameters of renal function, between the control and microcystin-LR-administrated rats, *prorenin* expression was significantly increased in the renal cortex of the rats administered microcystin-LR and the microcystin-LR-treated proximal tubular cells. The expression levels of *(pro)renin receptor* (*PRR*), *transforming growth factor-β1* (*TGFβ1*), and *α-smooth muscle actin* (*α-SMA*) in the renal cortex did not differ significantly between the control and microcystin-LR-administered rats. However, the expression levels of *prorenin* were significantly positively correlated with those of *PRR*, *TGFβ1*, and *α-SMA* in the renal cortex of rats administered microcystin-LR. Additionally, a significant positive correlation was observed between the expression levels of *TGFβ1* and *α-SMA*. Collectively, increased *prorenin* expression caused by the long-term consumption of microcystin-LR may initiate a process that influences renal fibrosis and abnormal renal function by regulating the expression levels of *PRR*, *TGFβ1*, and *α-SMA*.

## 1. Introduction

The development of harmful algal blooms in surface waters is caused by eutrophication [1]. Eutrophication is often associated with cyanobacterial species such as *Anabaena*, *Anabaenopsis*, *Aphanocapsa*, *Cichlidium*, *Fischerella*, *Gloeotrichia*, *Microcystis*, *Nodularia*, *Nostoc*, *Oscillatoria*, and *Planktothrix* [2,3,4,5,6]. Additionally, *Spirulina*, *Synechococcus*, and *Trichodesmium* have been identified as marine cyanobacteria [7,8,9,10,11]. The exponential growth in cyanobacteria leads to the production of secondary metabolites, specifically cyanotoxins [4,5,6,12]. Microcystins are a type of cyanotoxin that are found all over the world and have become a major health issue globally. These toxins are produced by cyanobacteria and are the only ones for which the World Health Organization (WHO) has established specific guidelines for safe drinking water levels [5,6]. There are 279 different types of microcystins, of which microcystin-LR is the most studied and the most commonly identified in the environment [4,5,6]. This particular microcystin variant makes up 46–99.8% of the total microcystin concentration in natural waters [13,14].

To evaluate the distribution of microcystin-LR in the body, ^125^I-microcystin-LR was injected intravenously, intraperitoneally, and orally into mice, and microcystin-LR was mainly distributed in the blood, liver, and kidneys [15]. In addition, Wistar rats intravenously injected with extracted microcystins showed higher levels of microcystins in the kidneys than in the liver [16]. These findings suggested that the kidney, as well as the liver, is a specific target organ for microcystin-LR. Epidemiological studies on nephrotoxicity induced by microcystins have documented a high prevalence of chronic kidney disease (CKD) in the Girandurukotte region of Sri Lanka, possibly because of microcystin contamination of well water [17,18]. A positive correlation between serum microcystins and abnormal renal function indicators among fishermen in Lake Chaohu, China, suggested that microcystins are involved in abnormal renal function [18,19]. A cross-sectional study conducted in Southwest China suggested that consumption of drinking water and aquatic products is one of the significant risk factors for abnormal renal function [18,20]. The above reports suggest that the consumption of drinking water and aquatic products contaminated with microcystin-LR is one of the most significant risk factors for abnormal renal function.

Renal fibrosis, characterized by tubulointerstitial fibrosis, in which the renal tubules undergo fibrosis, and which is recognized as the terminal stage in the development of end-stage kidney disease, is a crucial contributor to the pathogenesis of CKD [21,22,23]. Among the various mediators of renal fibrosis, transforming growth factor-β1 (TGFβ1) is a representative molecule that plays a central role in this process [23,24], particularly by activating the promoter region of α-smooth muscle actin (α-SMA) in renal fibrosis [25], resulting in increased α-SMA expression [25,26]. The induction of TGFβ1 is thought to be mediated by intrarenal activation of the renin–angiotensin system, which consists of angiotensinogen (AGT), the precursor of angiotensin II (Ang II), and the proteases renin and angiotensin-converting enzyme (ACE), along with associated locally produced Ang II [23,27]. However, there is also an alternative Ang II-independent pathway that contributes to renal fibrosis by involving the renin–angiotensin system. Prorenin, a precursor of renin, enhances its activity by binding to the (pro)renin receptor (PRR), and is not only involved in increasing the production of Ang I, a precursor of Ang II [28], but also promotes fibrosis of rat proximal tubular cells without angiotensin II signaling [29]. In addition, transgenic rats overexpressing human PRR show increased activation of mitogen-activated protein kinases (MAPs) and TGFβ1 expression, independent of AngII signaling [30]. This implies that the increased intracellular signaling pathway triggered by prorenin can contribute to renal fibrosis. Therefore, increased renal prorenin expression is considered to upregulate TGFβ1 and α-SMA expression by activating their respective intracellular signaling pathways through Ang II production and binding to PRR. That is, there is a strong possibility that the expression levels of prorenin, TGFβ1, and α-SMA are positively correlated.

Most previous reports on microcystin-LR in the kidney using rodents have relied on data from intraperitoneal injections [18,31,32]. However, an intraperitoneal injection is a route of exposure that is not representative of normal environmental conditions, since exposure to microcystins usually occurs through consuming contaminated drinking water and eating aquatic plants, fish, crops, and vegetables [33,34,35]. One non-intraperitoneal study reported on the oral administration of microcystin-LR to mice for 3 and 6 months. This study focused on renal function indices and histopathological observations of the kidneys, with no discussion of the underlying molecular mechanisms [18,36]. The transition from healthy kidneys to the onset of abnormal renal function is thought to be preceded by changes in gene expression prior to the onset of plasma parameters indicative of abnormal renal function and the emergence of histological modifications in the kidney. For instance, although previous findings have shown that albuminuria precedes the onset of abnormal renal function in patients with diabetes [37], prorenin expression is sometimes elevated before albuminuria is detected [38,39,40,41]. That is, it is possible for renal prorenin expression to increase even in the absence of any abnormalities in the plasma parameters that reflect renal function. Furthermore, increased prorenin expression has been found to be correlated with the development and progression of diabetic nephropathy [38,39,40,41]. Based on the above, rats orally administered microcystin-LR for 7 weeks, approximately half of the previously reported 3-month treatment period [18,36], were expected to show changes that would help to elucidate the molecular mechanisms involved in the progressive decline of renal function.

To explore the potential action mechanism of microcystin-LR in the kidney, the present study aimed to analyze whether rats orally administered microcystin-LR for 7 weeks would show changes in the expression of renal renin–angiotensin system-related and fibrosis-related genes involved in abnormal renal function. The concentrations of plasma albumin, blood urea nitrogen (BUN), and creatinine, indicators of kidney function, were also evaluated.

## 2. Materials and Methods

### 2.1. Materials and Reagents

Microcystin-LR, aprotinin, penicillin–streptomycin solution (×100), ITS-G Supplement (×100), and Dulbecco’s modified Eagle’s medium (DMEM)/F12 (Wako Pure Chemical Industries Ltd., Osaka, Japan); Heparin (Nacalai Tesque Inc., Kyoto, Japan); Trypsin-EDTA (Grand Island, NY, USA); Fetal bovine serum (FBS; Biosera, Nuaillé, France).

### 2.2. Animal Experiments

The Animal Care and Use Committee of Shimane University approved all animal experiments and procedures (protocol codes: MA28-1 and MA31-3), which were executed in accordance with the Institutional Regulations of Shimane University and complied with the Act on Welfare and Management of Animals (Act No. 105) and relevant standards and guidelines in Japan. Since the levels of female hormones in the blood vary among individuals based on their menstrual cycles, it is possible that the levels of other hormones in the blood and the function of organs and tissues may also exhibit individual differences. Therefore, males with no menstrual cycle were used in the present study. Five-week-old male wild-type (WKAH/HkmSlc) rats (Japan SLC, Inc., Hamamatsu, Japan) were housed individually in plastic cages in an air-conditioned room maintained at 22 ± 2 °C with 55 ± 5% humidity under an automated light cycle (lights on at 08:00 and off at 20:00). The rats were fed an AIN-93G diet without *t*-butylhydroquinone and deionized water ad libitum. After a week of acclimation, the rats were divided into the control and experimental groups, each comprising 11 individuals, and provided with unrestricted access to either deionized water (control group) or microcystin-LR (10 μg/L) in deionized water for 7 weeks [42]. The concentration of microcystin-LR utilized in this study corresponded to the highest level of microcystin detected in the final drinking water at the Celina plant, and in plants grown for consumption that received water sourced from Lake Erie and other locations in Ohio and neighboring states [4]. Following the treatment period, the abdominal aorta blood of rats was collected using a syringe containing heparin (final concentration of 50 U/mL blood) and aprotinin (final concentration of 500 kIU/mL blood) under anesthesia with 5% isoflurane for induction and 2% for maintenance via a nose cone. Plasma was prepared by centrifuging the collected abdominal aorta blood at 2000× *g* for 10 min at 4 °C. Prepared plasma and collected kidneys were stored at −80 °C until analysis.

### 2.3. Measurements of Albumin, Blood Urea Nitrogen, and Creatinine

Plasma concentrations of albumin, BUN, and creatinine were measured by Oriental Yeast Co., Ltd. (Tokyo, Japan).

### 2.4. Cell Culture

HK-2 cells derived from human proximal tubular cells (ATCC, Manassas, VA, USA) were cultured according to previously established protocols [26,43]. Briefly, the HK-2 cells were grown in DMEM/F12 medium supplemented with 10% FBS, ITS-G Supplement, 100 U/mL penicillin, and 100 μg/mL streptomycin at 37 °C in a 5% CO_2_ atmosphere. The cells were seeded (3 × 10^5^/dish) in 3.5 cm dishes and then incubated in serum-free DMEM/F12 for 24 h prior to all experiments. Dimethyl sulfoxide (DMSO, final concentration of 0.1% *v*/*v*) was used as the control and vehicle for microcystin-LR.

### 2.5. Quantitative Real-Time PCR

Serum-starved HK-2 cells were stimulated with or without microcystin-LR (10 nM) for various durations. Total RNA was extracted from the HK-2 cells and the kidneys were scraped using Sepasol-RNA I Super G (Nacalai Tesque Corporation, Kyoto, Japan) and RNeasy Mini Kits (QIAGEN, Hilden, Germany), respectively. For subsequent manipulations, gene expression levels were quantitatively assessed using established methods [44,45,46]. The oligonucleotide primers that were used are listed in Table 1. Amplicons were quantified based on a calibration curve of known DNA concentrations, and quantitation cycle (Cq) values were plotted against log sample concentrations. The mRNA expression was determined as the ratio of rat or human *ribosomal protein lateral stalk subunit P0* (*RPLP0*) mRNA, which served as an internal standard.

### 2.6. Statistical Analysis

**Figure 1:**
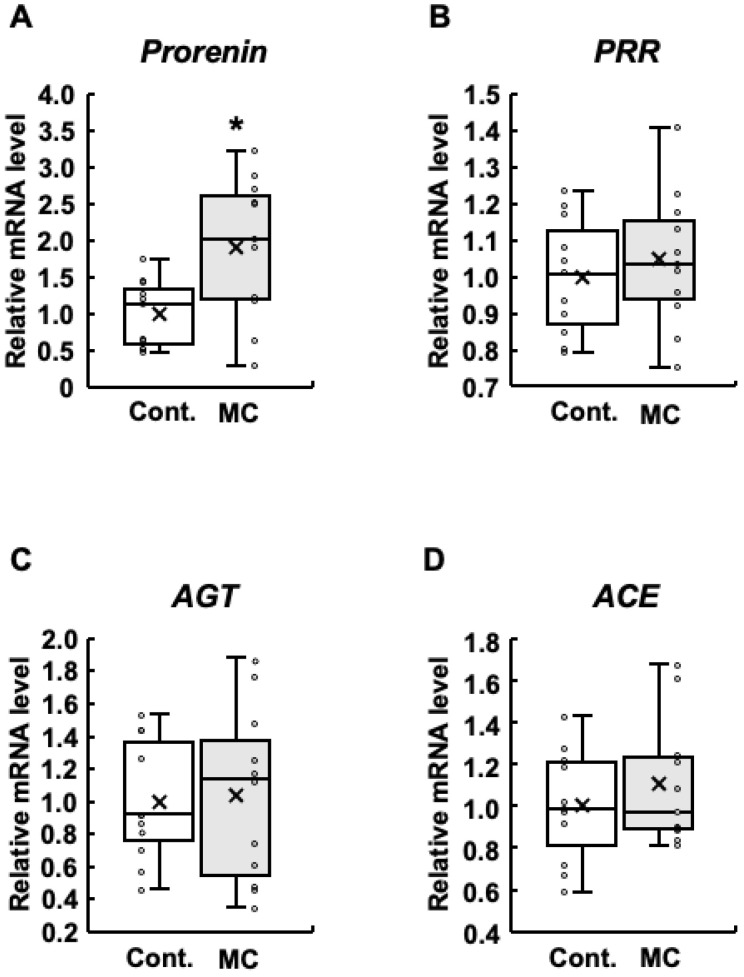
Expression of renin–angiotensin system-related genes in the renal cortex of rats. The expression of prorenin (**A**), PRR (**B**), AGT (**C**), and ACE (**D**) in the renal cortex was assessed using real-time PCR. Boxplots, which display the average, median, 25th, and 75th percentiles as boxes and the minimum and maximum values as whiskers, were used to show the results for both the control and microcystin-LR-administered rats. Cont.: control (*n* = 11); MC: microcystin-LR (*n* = 11); Circle: individual mRNA level; Cross: mean value. * *p* < 0.05 vs. control.

**Figure 2:**
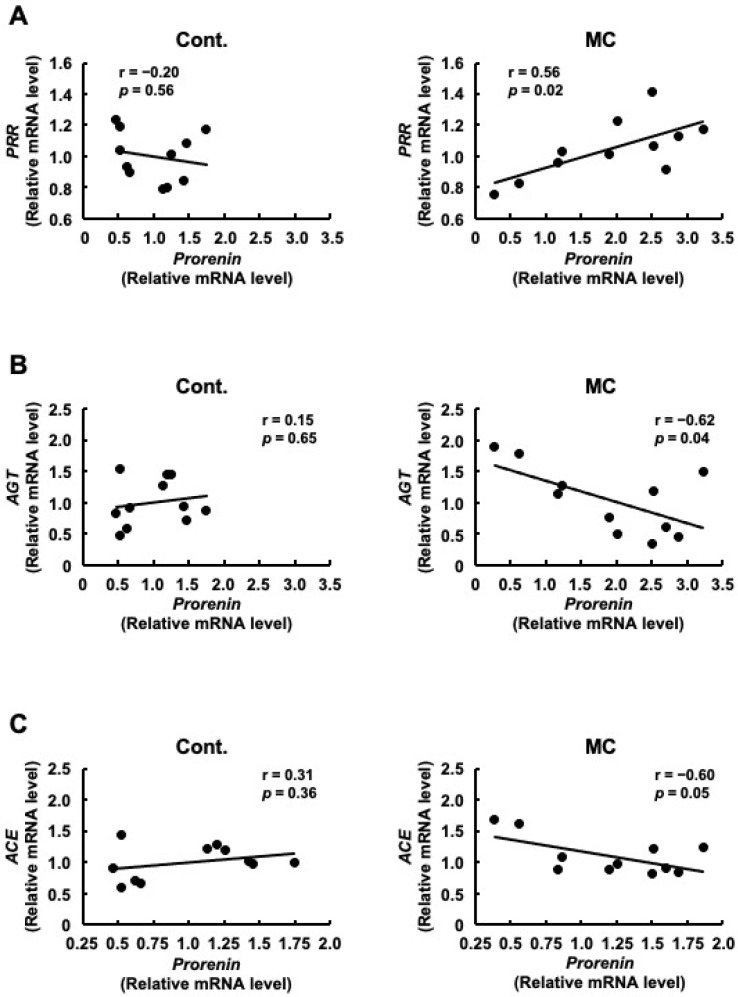
Correlation between the mRNA expression levels of prorenin and PRR, AGT, or ACE in the renal cortex of rats. The associations between prorenin and PRR (**A**), AGT (**B**), or ACE (**C**) expression were examined using Pearson’s correlation tests. Cont.: control (*n* = 11), MC: microcystin-LR (*n* = 11); Black dot: individual mRNA level.

**Figure 3:**
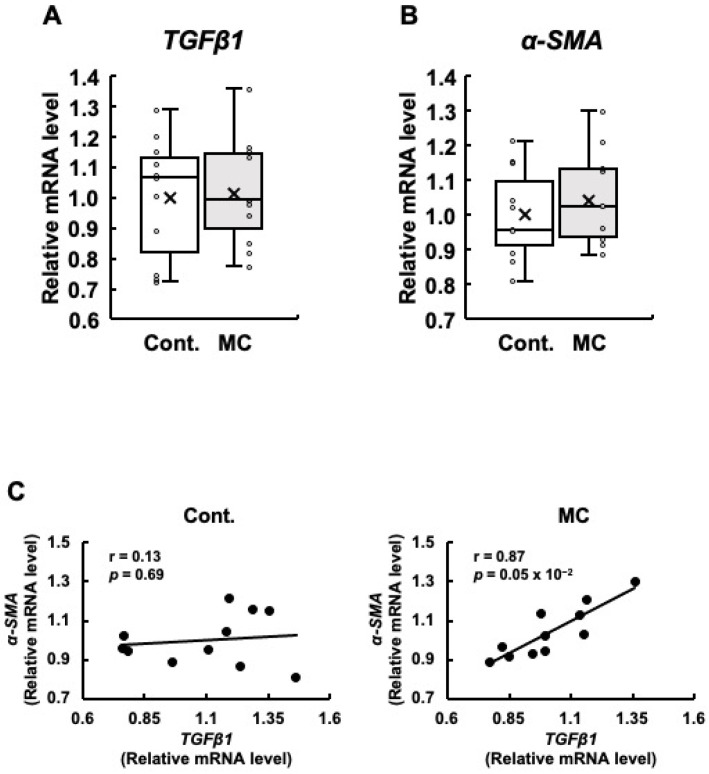
The expression of TGFβ1 and α-SMA mRNA in the renal cortex of rats, along with the correlation between their mRNA levels. The expression of TGFβ1 (**A**) and α-SMA (**B**) in the renal cortex was assessed using real-time PCR. Boxplots, which display the average, median, 25th, and 75th percentiles as boxes and the minimum and maximum values as whiskers, were used to show the results for both the control and microcystin-LR-administered rats. (**C**) The associations between TGFβ1 and α-SMA expression were examined using Pearson’s correlation tests. Cont.: control (*n* = 11); MC: microcystin-LR (*n* = 11); Circle: individual mRNA level; Cross: mean value; Black dot: individual mRNA level.

**Figure 4:**
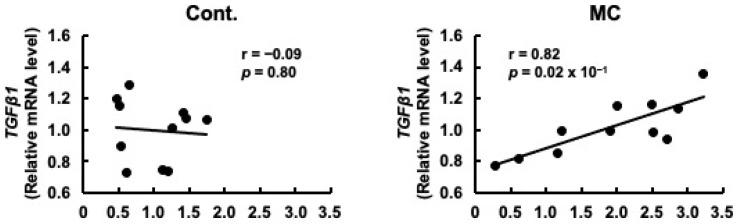
Correlation between the mRNA expression levels of *prorenin* and *TGFβ1* in the renal cortex of rats. The associations between *prorenin* and *TGFβ1* expression were examined using Pearson’s correlation tests. Cont.: control (*n* = 11); MC: microcystin-LR (*n* = 11); Black dot: individual mRNA level.

**Figure 5:**
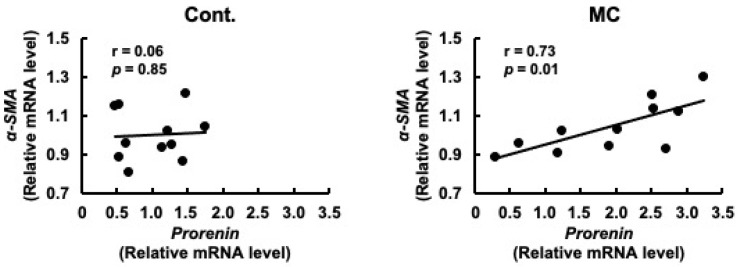
Correlation between the mRNA expression levels of *prorenin* and *α-SMA* in the renal cortex of rats. The associations between *prorenin* and *α-SMA* expression were examined using Pearson’s correlation tests. Cont.: control (*n* = 11); MC: microcystin-LR (*n* = 11); Black dot: individual mRNA level.

**Figure 6:**
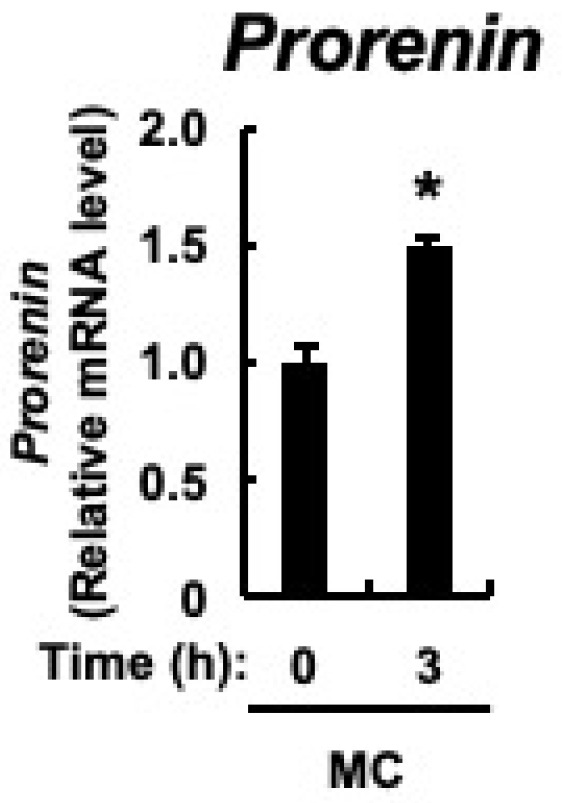
Expression of *prorenin* in HK-2 cells. HK-2 cells deprived of serum were cultured for varying periods with or without microcystin-LR (10 nM). *Prorenin* expression was measured using real-time PCR. The data, expressed as the mean ± SE of *n* = 3, showed an increase in mRNA expression in the presence of microcystin-LR. MC: microcystin-LR. * *p* < 0.05 vs. 0 h.

The results were reported as the mean ± standard error (SE) because SE allows for interval estimation of the population mean. Data were analyzed for normality, followed by the Mann–Whitney U test if they did not follow a normal distribution (Table 2 [kidney weight and albumin] and Figure 1D). Conversely, when the data followed a normal distribution, the Student’s *t*-test was used when the variances of the two groups were assumed to be equal (Table 2 [BUN and creatinine], Figure 1B,C, Figures 3A,B, and 6). On the other hand, the Welch’s *t*-test was employed when the variances of the two groups were assumed to be unequal (Figure 1A). Pearson correlations were used for correlation analysis (Figures 2A–C, 3C, 4, and 5). Statistical analyses were performed using Excel 2011 (Microsoft Corp., Redmond, WA, USA) and Statcel 4 (OMS Publishing Co., Saitama, Japan). Statistical significance was set at *p* < 0.05.

## 3. Results

### 3.1. The Effect of Administering Microcystin-LR to Rats for 7 Weeks on Renal Function

Using rats that were noninvasively exposed to environmentally relevant concentrations of microcystin-LR (10 µg/L) [4] in ad libitum drinking water for 7 weeks [42], the present study examined the impact on renal status. In addition to the lack of differences in body weight and food intake between the control and microcystin-LR-administered rats [39], there were no differences in the kidney weight and the concentrations of plasma albumin, BUN, and creatinine, which are parameters of renal function, between the two groups (Table 2). These results indicated that prolonged environmental exposure to microcystin-LR for 7 weeks does not induce significantly abnormal renal function.

### 3.2. The Influence of Administering Microcystin-LR to Rats for 7 Weeks on the Expression Levels of Genes Associated with the Renin–Angiotensin System in the Renal Cortex

In patients with diabetes, increased prorenin expression appears prior to the onset of albuminuria, which is typically observed before a decline in renal function and is positively associated with the development and progression of diabetic nephropathy [38,39,40,41]. In addition, increased expression of renin–angiotensin system-related genes is involved in abnormal renal function [23]. Based on these reports, we examined the expression levels of *prorenin*, *PRR*, *AGT*, and *ACE* in the renal cortex. The expression levels of *prorenin* were significantly increased in rats orally administered microcystin-LR compared to those in the control group (Figure 1A). In addition, the maximum, upper quartile, median, lower quartile, and mean values were also higher in the rats orally administered microcystin-LR (Figure 1A). The results for *PRR* were similar to those for prorenin, although no significant increase in expression levels was observed (Figure 1B). *AGT* had higher maximum and median values (Figure 1C), and *ACE* had higher maximum, minimum, and mean values in rats orally administered microcystin-LR (Figure 1D). Taken together, *prorenin* expression was significantly and markedly increased in the renal cortex of rats orally administered microcystin-LR.

### 3.3. The Correlation between the Expression Levels of Prorenin and PRR, AGT, or ACE Following Microcystin-LR Administration to Rats for 7 Weeks

Although there was no significant difference in *PRR* expression between the control and microcystin-LR-administered rats, the pattern of *PRR* expression was similar to that of *prorenin* expression (Figure 1). Therefore, we examined the correlation between the expression levels of *prorenin* and other renin–angiotensin system-related genes, including *PRR*. Figure 2A shows that the expression levels of *prorenin* and *PRR* were significantly and positively correlated in the renal cortex of rats orally administered microcystin-LR (*r* = 0.56, *p* = 0.02). In contrast, the expression levels of *prorenin* and *AGT* were significantly and inversely correlated in the renal cortex of rats orally administered microcystin-LR (*r* = −0.62, *p* = 0.04) (Figure 2B). The expression levels of *prorenin* and *ACE* tended to be inversely correlated in the renal cortex of rats orally administered microcystin-LR (*r* = 0.60, *p* = 0.05) (Figure 2B). Taken together with the report that prorenin induces increased PRR expression in rat renal proximal tubule cells [29], these results suggested that prolonged environmental exposure to microcystin-LR is involved in changing the regulation of intracellular gene expression by increasing *prorenin* expression, which may lead to an increase in *PRR* expression in the future. In addition, elevated *prorenin* expression and prolonged environmental exposure to microcystin-LR might also affect the expression levels of *AGT* and *ACE* in rats orally administered microcystin-LR.

### 3.4. The Impact of Administering Microcystin-LR to Rats for 7 Weeks on the Expression Levels of Genes Associated with Fibrosis, as Well as the Relationship between the Expression Levels of These Genes in the Renal Cortex

Since renal fibrosis is an important contributor to the progression of abnormal renal function, we examined the expression levels of *TGFβ1* and *α-SMA*, which are both linked to renal fibrosis. Our analysis did not reveal any statistically significant differences in the expression levels of *TGFβ1* and *α-SMA* between the control and microcystin-LR-administered rats (Figure 3A,B). However, the rats orally administered microcystin-LR displayed increased maximum, upper quartile, lower quartile, and minimum values for the expression levels of *TGFβ1* (Figure 3A), and increased maximum, upper quartile, median, lower quartile, minimum, and mean values for the expression levels of *α-SMA* (Figure 3B). Even if the *p*-value of the *t*-test was 0.05 or higher, if the maximum, minimum, upper, and lower quartiles, and mean values of the boxplots were all higher in the microcystin-LR-administered rats than in the control rats, as in the present study, it was possible that gene expression in the renal cortex of the rats orally administered microcystin-LR may have begun to change. In particular, TGFβ1 is implicated in the upregulation of α-SMA expression [26]. Based on this finding, if the expression levels of these two genes are correlated, this result suggests that this is a process in which the activation of the TGFβ1/α-SMA signaling pathway begins to increase. The results in Figure 3C show a significant and positive correlation between the expression levels of *TGFβ1* and *α-SMA* in the renal cortex of rats orally administered microcystin-LR (*r* = 0.87, *p* = 0.05 × 10^−2^). This was not observed in the control rats. These results suggested that the regulatory mechanisms of gene expression that induce renal fibrosis begin to operate in rats orally administered microcystin-LR.

### 3.5. Relationship between the Expression Levels of Renin–Angiotensin System-Related Genes and Fibrosis-Related Genes in the Renal Cortex of Rats Following Microcystin-LR Administration for 7 Weeks

Prorenin has been suggested to contribute to the increased expression of fibrosis-related genes in rat and human proximal tubular cells, and in rats with CKD [29,47]. Based on these reports, we predicted that increased *prorenin* expression due to prolonged environmental exposure to microcystin-LR may alter the regulation of intracellular gene expression and begin to affect the expression levels of renal *TGFβ1* and *α-SMA*. Therefore, we assessed whether there was a correlation between the expression levels of *prorenin* and those of fibrosis-related genes. The expression levels of prorenin and *TGFβ1* were significantly and positively correlated in the renal cortex of rats orally administered microcystin-LR (*r* = 0.82, *p* = 0.02 × 10^−1^) (Figure 4). In addition, the expression levels of *prorenin* and *α-SMA* were significantly and positively correlated in the renal cortex of the rats orally administered microcystin-LR (*r* = 0.73, *p* = 0.01) (Figure 5). Taken together, the relationship between the expression levels of *prorenin* and fibrosis-related genes was observed only in the renal cortex of rats orally administered microcystin-LR. Therefore, changes in the regulation of intracellular gene expression due to increased *prorenin* expression induced by prolonged environmental exposure to microcystin-LR may upregulate the expression of renal fibrosis-related genes.

### 3.6. Effects of Microcystin-LR on the mRNA Expression Levels of Prorenin in Cultured Proximal Tubular Cells

The major cellular components of the renal cortex are the renal tubules, and prorenin expression is upregulated in the renal tubular cells of rats with CKD [43]. In cultured proximal tubular cells, prorenin expression is increased by the accumulation of indoxyl sulfate in the blood of patients with CKD [47]. Therefore, we examined whether microcystin-LR, as well as indoxyl sulfate, would result in increased *prorenin* expression in proximal tubular cells. Microcystin-LR concentrations in urine collected 24 h after intraperitoneal administration of microcystin-LR at a dose of 10 μg/kg to male mice were reported to be up to approximately 10 ug/L [48]. Since the molecular weight of microcystin-LR is 995.17, a cultured proximal tubular cell line, HK-2 cells, was stimulated with 10 nM microcystin-LR, which is equivalent to 10 μg/mL. As shown in Figure 6, in addition to upregulation of *prorenin* expression, there was already a significant increase in its expression 3 h after microcystin-LR stimulation. This indicated that *prorenin* expression may be upregulated by microcystin-LR in the proximal tubular cells of the renal cortex.

## 4. Discussion

Although prolonged environmental exposure to microcystin-LR may cause abnormal renal function, the specific sites and genes responsible for this effect are not yet fully understood. *Prorenin* expression was significantly upregulated in the renal cortex of rats orally administered microcystin-LR and in proximal tubular cells treated with microcystin-LR. Significant positive correlations were observed between the expression levels of prorenin and *PRR*, *TGFβ1*, or *α-SMA* in the renal cortex of rats orally administered microcystin-LR. In addition to prorenin leading to increased expression of PRR and fibrosis-related genes in rat proximal tubular cells [29], prorenin, PRR, TGFβ1, and α-SMA are increased in the renal tubules of rats with CKD [47,49,50]. Based on these reports, prolonged environmental exposure to microcystin-LR could lead to increased *prorenin* expression in the renal tubules, ultimately resulting in abnormal renal function via renal fibrosis. Furthermore, increased *PRR* expression, associated with increased prorenin expression, may induce renal fibrosis through amplified activation of Wnt/β-catenin signaling by prorenin-independent PRR [51], in addition to enhanced activation of the renin–angiotensin system and MAPKs [28,30]. Therefore, the present findings provide important mechanistic insights into the development and progression of abnormal renal function due to prolonged environmental exposure to microcystin-LR, with increased *prorenin* expression representing the first step in this process.

Increased prorenin expression is sometimes observed before the onset of albuminuria and the elevation of plasma parameters indicating abnormal renal function in patients with diabetes [38,39,40,41]. In addition, increased prorenin expression correlates with the onset and progression of diabetic nephropathy [38,39,40,41]. Prolonged environmental exposure to microcystin-LR for 7 weeks did not change the plasma parameters indicating abnormal renal function, but increased *prorenin* expression was observed. Therefore, prolonged environmental exposure to microcystin-LR over a long period of time may eventually lead to abnormal renal function, similar to in diabetes. Prorenin may therefore be a marker for predicting abnormal renal function due to prolonged environmental exposure to microcystin-LR, as observed in patients with diabetes. However, because prorenin expression is already elevated in patients with diabetes, it is difficult to predict the abnormal renal function induced by prolonged environmental exposure to microcystin-LR using increased prorenin expression as an index in such patients.

Expression levels of prorenin were positively correlated with those of the fibrosis-related genes *TGFβ1* and *α-SMA* in rats orally administered microcystin-LR for 7 weeks. Therefore, the mechanism of early renal fibrosis induced by prolonged environmental exposure to microcystin-LR may be affected not only by the intracellular signaling pathway from Ang II generated from AGT, but also by the activation of the intracellular signaling pathway by increasing prorenin expression. In fact, treatment of rat proximal tubular cells with ARB followed by prorenin stimulation increases the expression of fibrosis-related genes in a dose- and time-dependent manner [29]. In addition, in transgenic rats overexpressing human PRR, in which prorenin signaling is assumed to be enhanced, TGFβ1 expression is increased despite a significant decrease in renal Ang II levels due to ACEI [30]. In addition to the above correlations, in rats orally administered microcystin-LR for 7 weeks, the expression levels of *prorenin* and *PRR* were positively correlated, and the expression levels of *PRR* were positively correlated with those of *TGFβ1* and *α-SMA* (Figure S1). PRR may not only enhance prorenin-dependent activation of the renin–angiotensin system and MAPKs [28,30], but may also induce renal fibrosis through amplified activation of Wnt/β-catenin signaling by a prorenin-independent PRR mechanism [51]. Via mechanisms other than the above, microcystin-LR may enhance activation of the TGFβ1/α-SMA signaling pathway. TGFβ1 causes receptor-mediated serine phosphorylation of Smad2/3, and serine phosphorylated Smad2/3 forms a complex with Smad4, thereby binding to the promoter region of α-SMA [25]. Since microcystin-LR has a specific affinity for and inhibits serine/threonine protein phosphatases 1 and 2A [4,5,6,18], increased expression of α-SMA may be enhanced in rats orally administered microcystin-LR for long periods of time, due to the enhanced serine oxidation of Smad2/3 induced by TGFβ1 stimulation. This effect may occur independently of increased prorenin expression. The primary therapeutic targets for the management of patients with CKD are generally considered to be inhibition of Ang II production with ACE inhibitors (ACEIs) or inhibition of Ang II signaling with angiotensin II type 1 receptor blockers (ARBs) [23]. However, based on the present findings, ACEIs and ARBs may not be effective for early abnormal renal function owing to prolonged environmental exposure to microcystin-LR.

PRO20, the initial 20 amino acid residues of the prorenin prosegment L1PTDTASFGRILLKKMPSVR20, serves as a novel antagonistic peptide that effectively impedes the interaction between prorenin and PRR [52]. Therefore, the binding of prorenin and PRR, blocked by PRO20, is expected to suppress the progression of renal fibrosis and abnormal renal function through inhibiting activation of the renin–angiotensin system to produce Ang II and prorenin/PRR-dependent intracellular signaling pathways. The expression levels of prorenin, PRR, TGFβ1, and α-SMA are all upregulated in rats with CKD [47,49,50]. Administration of PRO20 in similar rats with CKD ameliorated urinary/renal levels of renin activity, AGT, and Ang II, as well as inhibiting Wnt/β-catenin signaling. Finally, administration of PRO20 greatly suppresses renal fibrosis and abnormal renal function in rats with CKD [53]. Pathological lesions have been observed in the kidneys of mice orally administered microcystin-LR for 3 and 6 months [18,36]. In addition, the results obtained in the present study indicated that when microcystin-LR is administered to rats chronically for a long period of time, the expression levels of *PRR*, *TGFβ1*, and *α-SMA* are likely to increase significantly with an increase in *prorenin* expression compared with control rats. Based on the above findings, PRO20 appears to be highly effective in preventing the progression of renal fibrosis and abnormal renal function caused by prolonged environmental exposure to microcystin-LR. Thus, it will be necessary to analyze in the future whether administration of microcystin-LR with PRO20 for an even longer period would prevent the development and progression of renal fibrosis associated with abnormal renal function via PRR in rats. In addition, since increased prorenin expression is predicted to promote activation of the renin–angiotensin system, an elevation in blood pressure should also be noted.

The present study showed that the expression levels of *prorenin* were negatively correlated with those of *AGT* in the renal cortex of rats orally administered microcystin-LR. These results were probably due to the fact that rats orally administered microcystin-LR for 7 weeks did not show any decline in their renal functions, as indicated by the results of the plasma parameters that are indicators of renal function. When CKD is caused by abnormal renal function, indoxyl sulfate accumulates in the blood of both humans and rats with the progression of the disease [54,55,56,57,58], thereby upregulating the expression levels of AGT, as well as prorenin, in the renal cortex [43,59]. Therefore, if the rats were orally administered microcystin-LR for more than 7 weeks, leading to a decrease in renal function, the expression levels of *prorenin* would be predicted to positively correlate with those of *AGT*, since indoxyl sulfate is expected to accumulate in the blood, similar to in rats with CKD.

Microcystins have been detected in closed water systems in many parts of the world in recent years. In the summer of 2018, 28 out of the 30 subtropical lakes in eastern China were found to contain microcystins, with Chaohu Lake recording the highest average concentration of microcystins at 26.7 μg/L [60]. In addition, according to a recent survey of 24 drinking water treatment plants in the United States, 75% of samples tested positive for microcystin-LR contamination. Some of these samples contained microcystin-LR concentrations that were not safe for human consumption [4]. In Europe, microcystins constitute approximately 60% of the detectable cyanotoxins present in brackish and freshwater systems [61]. Against this background, it is important to develop methods to detect the dysfunction of organs and tissues induced by microcystins in advance. We believe that the increased prorenin expression observed in the present study can be used as a predictive biomarker for the onset and progression of abnormal renal function induced by microcystins through future studies. However, the current study exclusively used male rats for the analysis. Therefore, future analyses using female rats should be conducted to determine whether increased prorenin expression is observed upon prolonged environmental exposure to microcystin-LR across the sexes.

## 5. Conclusions

Prolonged environmental exposure to microcystin-LR is considered to cause renal fibrosis and abnormal renal function due to increased *prorenin* expression in the proximal tubular cells of the renal cortex. Therefore, the identification of dietary components and the development of therapeutic agents that inhibit elevated *prorenin* expression may be important for suppressing the onset and progression of renal fibrosis and abnormal renal function induced by the continuous consumption of microcystins. Furthermore, since prorenin may serve as a useful biomarker for predicting the onset and progression of renal fibrosis and abnormal renal function induced by the continuous consumption of microcystins in patients other than those with diabetes who already have elevated prorenin expression, using prorenin as an indicator may help prevent the onset and progression of CKD induced by the continuous consumption of microcystins.

## Figures and Tables

**Table 1:** Forward (Fw) and reverse (Rv) primers for target genes.

Target Genes	GenBank Accession No.	Primers (5→3′)	Length (bp)	Product Length (bp)
**Rat**				
*Prorenin*	NM_012642.4	Fw: CTCTCTGGGCACTCTTGTTGC	21	198
		Rv: GGGAGGTAACATTGGTAAAGGA	22	
*PRR*	NM_001007091.1	Fw: TTCTGAACTGCAAGTGCTGCAT	22	101
		Rv: CTGCCAGCTCCAGTGAATACAAG	23	
*AGT*	NM_134432.2	Fw: GTGGAGGTCCTCGTCTTCCA	20	108
		Rv: GTTGTAGGATCCCCGAATTTCC	22	
*ACE*	NM_012544.1	Fw: TGGGGACAAATACATCAATCTCA	23	105
		Rv: GGGAAAGGCACTACCATGTCG	21	
*TGFβ1*	NM_021578.2	Fw: AGCTGGTGAAACGGAAGCG	19	64
		Rv: GCGAGCCTTAGTTTGGACAGG	21	
*α-SMA*	NM_031004.2	Fw: GTCCCAGACACCAGGGAGTGA	21	102
		Rv: TCGGATACTTCAGGGTCAGGA	21	
*RPLP0*	NM_022402.2	Fw: GCTCCAAGCAGATGCAGCA	19	143
		Rv: CCGGATGTGAGGCAGCAG	18	
**Human**				
*Prorenin*	NM_000537.4	Fw: CCGTGATCCTCACCAACTACA	21	112
		Rv: ACCCAAACATTGGACGAACCA	21	
*RPLP0*	NM_001002.4	Fw: CGACCTGGAAGTCCAACTAC	20	108
		Rv: ATCTGCTGCATCTGCTTG	18	

**Table 2:** Kidney weights and plasma parameters in the control and microcystin-LR-administered rats after 7 weeks.

	Control	Microcystin-LR	*p*
Kidney weight (g/100 g body weight)	0.53 ± 3.40	0.55 ± 3.34	*NS*
Albumin (g/dL)	3.3 ± 0.07	3.3 ± 0.02	*NS*
Blood urea nitrogen (mg/dL)	17.4 ± 0.85	16.5 ± 0.57	*NS*
Creatinine (mg/dL)	0.4 ± 0.01	0.3 ± 0.02	*NS*

Values are shown as the mean ± SE (*n* = 11 each). NS: not significant.

## Data Availability

The data presented in this study are available on request from the corresponding author.

## Supplementary Materials

The following supporting information can be downloaded at: https://www.mdpi.com/article/10.3390/toxics12080547/s1, Figure S1: Correlation between the mRNA expression levels of *PRR* and *TGFβ1* or *α-SMA* in the renal cortex of rats.
