# Supplementary figures and images for: Increased Prorenin Expression in the Kidneys May Be Involved in the Abnormal Renal Function Caused by Prolonged Environmental Exposure to Microcystin-LR
Source: Toxics. 2024 Jul 27;12(8):547. doi: 10.3390/toxics12080547 (PMC11360727; doi:10.3390/toxics12080547)

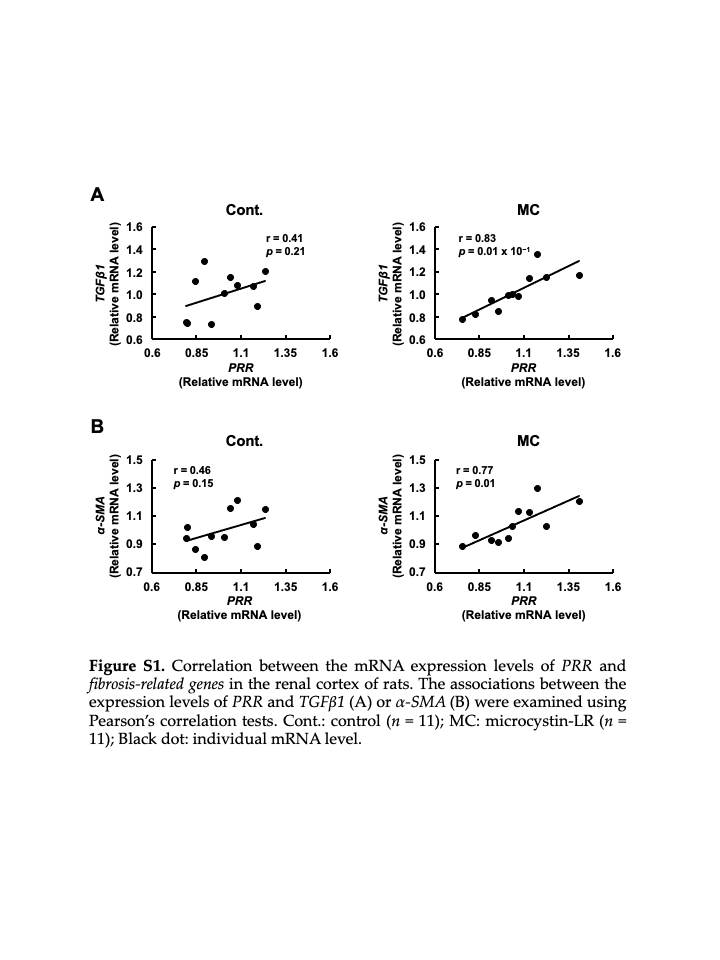

Supplement: Supplementary file 1 [file toxics-12-00547-s001.zip › toxics-3121354-supplementary.tiff]
